# Combination of preoperative plasma fibrinogen and AJCC staging improves the accuracy of survival prediction for patients with stage I‐II gastric cancer after curative gastrectomy

**DOI:** 10.1002/cam4.2086

**Published:** 2019-05-02

**Authors:** Peng Ding, Chen Zheng, Guohui Cao, Ziming Gao, Yuying Lei, Peng Deng, Bin Hou, Kai Li

**Affiliations:** ^1^ Department of Surgical Oncology and General Surgery The First Affiliated Hospital of China Medical University Shenyang China; ^2^ Department of Oncology The Hebei Province General Hospital Shijiazhuang China

**Keywords:** fibrinogen, gastric cancer, prognosis, staging

## Abstract

This study aimed to determine the prognostic value of preoperative plasma fibrinogen concentration (PFC) in patients with stage I‐II gastric cancer after curative gastrectomy. The preoperative PFC and clinicopathological data of 793 patients with stage I‐II gastric cancer who underwent curative gastrectomy were analyzed retrospectively. PFC of <4.0 g/L and ≥4.0 g/L were considered as PFC0 and PFC1, respectively. The association between PFC and the clinicopathological features of gastric cancer and the value of PFC in survival prediction were investigated. PFC1 indicated poorer overall survival and cancer‐specific survival among patients with tumor‐node‐metastasis (TNM) stage I‐II, and PFC was identified as an independent indicator of survival via multivariate analysis. Importantly, PFC stage was proven to be an independent prognostic factor for stage I and T1‐4aN0 gastric cancer. PFC stage combined with the American Joint Committee on Cancer (AJCC)‐TNM stage has better accuracy for predicting disease prognosis than AJCC‐TNM stage alone. The prognosis of patients with stage I‐II gastric cancer can be further stratified by PFC level. For patients with stage I gastric cancer, PFC1 can be considered a high‐risk prognostic factor, and adjuvant chemotherapy should be recommended for patients with PFC1.

## 1 INTRODUCTION

Gastric cancer (GC) is defined as the development of malignant cells in the stomach lining. GC has the highest incidence among all malignancies of the digestive system and is the second leading cause of cancer‐related death worldwide.^1^, ^2^ Although radical D2 gastrectomy has been the main treatment modality for GC, the mortality rate is still particularly high due to the high incidence of recurrence and metastasis after curative resection. An individualized treatment strategy based on survival prediction of a GC patient can improve overall outcomes. Adequate staging is critical to accurately predict prognosis, which is helpful for developing a comprehensive treatment plan. For instance, patients with stage II and III GC will benefit from adjuvant chemotherapy using a combination of capecitabine and oxaliplatin or S‐1 after D2 resection.^3^, ^4^ The American Joint Committee on Cancer (AJCC)‐TNM staging system for GC is considered the most accurate classification system to estimate prognosis and guide treatment based on the deep invasion of tumor (T stage), the extent of local nodal involvement (N stage), and the presence or absence of distant metastases (M stage). However, because GC is a systemic disease, micrometastases may be present in each period of tumor progression; thus, the accuracy of the TNM staging system combined with anatomical prognostic factors for estimating survival is limited. The prognosis of GC is also related to the tumor microenvironment and the interaction between the tumor and the host environment; hence, nonanatomical prognostic factors cannot be neglected. To date, numerous nonanatomical prognostic factors for predicting the prognosis of patients with GC have been evaluated, such as neutrophil‐lymphocyte ratio,^5^ Glasgow Prognostic Score,^6^ and albumin‐to‐globulin ratio.^7^

Although patients with GC with stage I and N0 generally have high survival rate after radical surgery, accurately predicting the overall outcomes for these patients is difficult because of the relatively low incidence of relapse or metastasis for stage I patients and the availability of only one prognostic indicator for node‐negative patients. Furthermore, whether adjuvant chemotherapy has a survival benefit for these patients remains unclear. Therefore, nonanatomical prognostic factors should also be considered to improve the prognostic accuracy of the AJCC‐TNM staging system and to further discriminate the prognostic risk in patients with stage I and N0 GC.

As a type of easily accessible nonanatomical prognostic indicator, plasma fibrinogen concentration (PFC) has been demonstrated to be associated with tumorigenesis, angiogenesis, and hematogenous metastasis of malignant tumor cells.^8^ Studies also showed that elevated preoperative PFC was independently correlated with poor prognosis in GC.^9^, ^10^, ^11^, ^12^, ^13^ However, no research has studied the significance of preoperative PFC as a prognostic predictor and used it to improve the accuracy of the factors for predicting prognosis after radical gastrectomy in patients with stage I and N0 GC. The purpose of this study was to investigate the prognostic stratification capability of PFC in patients with stage I‐II GC undergoing radical resection, particularly those with stage I and T1‐4aN0.

## 2 MATERIALS AND METHODS

### 2.1 Patients

We reviewed the records of consecutive patients who underwent gastrectomy for histologically diagnosed adenocarcinoma of the stomach between January 2005 and December 2012 at a single medical institution in China. Patients with chronic liver disease, distant or peritoneal metastasis, and coagulation disorders; those who received anticoagulation therapy, nonradical resection, preoperative transfusion, preoperative chemotherapy; or radiotherapy; those lost to follow‐up; and those who died within 30 days of surgery were excluded. The pathological tumor stage was evaluated using the eighth edition of the TNM‐Union for International Cancer Control/AJCC classification.

### 2.2 Follow‐up

All patients underwent a standardized follow‐up every 3 months for the first 2 years after the surgery, every 6 months in the third year, and yearly thereafter. The follow‐up period from the surgery lasted until the patient's death or December 2015 (for patients who underwent surgery between January 2005 and December 2010) or December 2017 (for patients who underwent between January 2011 and December 2012).

### 2.3 Blood sample collection

Levels of plasma fibrinogen, platelet, and hemoglobin were examined within 7 days before surgery. PFC was analyzed using French STAGO Magnetic Beads Coagulation detection and according to the manufacturer's instructions. PFC of <4.0 g/L was considered normal (PFC0), while PFC ≥4.0 g/L was considered as hyperfibrinogenemia (PFC1). Thrombocytosis was defined as platelet count of ≥300 × 109/L, and anemia was defined as hemoglobin <120 g/L in men and <110 g/L in women according to the normal reference range in our hospital.

### 2.4 Postoperative adjuvant chemotherapy

Patients with pT3‐4, pN (+), and pT2N0 with poorly differentiated or high‐grade cancer lymphovascular or neural invasion received the adjuvant therapy. Those who cannot endure chemotherapy were excluded from the treatment. Briefly, adjuvant chemotherapy was administered using tegafur gimeracil oteracil potassium capsule single drug, 5‐fluorouracil/cisplatin, 5‐fluorouracil/mitomycin/epirubicin, 5‐fluorouracil/leucovorin/cisplatin, 5‐fluorouracil/cisplatin/epirubicin, and cisplatin/oxaliplatin.

### 2.5 Statistical analysis

Pretreatment plasma fibrinogen levels were reported as the median ± interquartile range (IQR). Associations between PFC and clinicopathological factors were analyzed using the Kruskal‐Wallis or Mann‐Whitney U test for quantitative variables. The differences between categories of PFC and clinicopathological factors were compared using chi‐squared test. The rates of overall survival (OS) and cancer‐specific survival (CSS) were assessed via Kaplan‐Meier method, and comparisons between curves were conducted using the log‐rank test. Multivariate survival analysis was performed using the Cox proportional hazards regression model. All statistical analyses were performed using SPSS 22.0 statistical package (IBM, New York, USA), and two‐sided *P* < 0.05 was considered statistically significant.

The concordance index (C‐index), which was calculated using the package of Survival (http://CRAN.R-project.org/package=Survival) in R (version 3.4.1, http://www.R-project.org/), was used to compare the prognostic accuracy of different models.

## 3 RESULTS

### 3.1 Relationship between PFC and clinicopathological factors of patients with stage I‐II GC

Of the 1761 consecutive patients identified, 1472 were eligible for analysis. Among them, 793 patients with stage I‐II GC were enrolled in the current study. The median follow‐up period was 65 months (range, 2‐122 months). In total, 50 (6.3%) and 742 (93.7%) patients underwent D1/D1+ and D2/D2+ lymph node dissection, respectively. More than 16 lymph nodes were examined in each of the 680 (85.8%) patients. The median PFC in all patients with stage I‐II GC was 3.43 ± 1.22 g/L. The PFC in patients with late T and TNM stage was higher than in those with early stage (T1: 3.16 ± 0.97 vs T2: 3.38 ± 1.16 vs T3: 3.69 ± 1.29 vs T4: 3.70 ± 1.18, *P* = 0.000; IA: 3.17 ± 0.98 vs IB: 3.33 ± 1.19 vs IIA: 3.58 ± 1.31 vs IIB: 3.61 ± 1.18, *P* < 0.001). A total of 213 patients (28.66%) had PFC1. Late TNM stage was more common among the patients with PFC1 (IA: 16.1% vs IB: 28.1% vs IIA: 32.7% vs IIB: 34.0%, *P* < 0.001). The correlation between PFC level and the clinicopathological characteristics of the patients with stage I‐II GC is summarized in Table 1.

**Table 1:** Association of plasma fibrinogen with clinicopathologic factors in patients with stage I‐II gastric cancer

Factors	N	PFC <4 g/L (PFC0 stage)	PFC≧4 g/L (PFC1 stage)	*P*	PFC (g/L) (median ± IQR)	*P*
Gender						
Male	570 (71.9%)	410 (71.9%)	160 (28.1%)	0.219*	3.41 ± 1.26	0.615†
Female	223 (28.1%)	170 (76.2%)	53 (23.8%)		3.43 ± 1.08	
Age						
<60 y	449 (56.6%)	358 (79.1%)	91 (20.3%)	<0.001*	3.28 ± 1.03	<0.001†
≧60 y	344 (43.4%)	222 (64.5%)	122 (35.5%)		3.61 ± 1.25	
Tumor size						
<5 cm	571 (72%)	445 (77.9%)	126 (22.1%)	0.000*	3.30 ± 1.03	<0.001†
≧5 cm	222 (28.0%)	135 (60.8%)	87 (39.2%)		3.78 ± 1.25	
Tumor location						
Upper	76 (9.6%)	55 (72.4%)	21 (27.6%)	0.873*	3.40 ± 1.15	0.871†
Middle‐Lower	717 (90.4%)	525 (73.2%)	192 (26.8%)		3.43 ± 1.24	
Borrmann type						
Borrmann 1	30 (6.0%)	23 (76.7%)	11 (23.3%)	0.329*	3.51 ± 1.16	0.386†
Borrmann 2	61 (12.3%)	45 (73.8%)	16 (26.2%)		3.35 ± 1.00	
Borrmann 3	382 (76.9%)	248 (64.9%)	134 (35.1%)		3.61 ± 1.22	
Borrmann 4	24 (4.8%)	17 (70.8%)	7 (29.4%)		3.59 ± 1.48	
Differentiation						
High	112 (12.1%)	79 (70.5%)	33 (29.5%)	0.138*	3.54 ± 1.31	0.026†
Moderate	263 (33.2%)	184 (74.0%)	79 (30.0%)		3.54 ± 1.28	
Poor	233 (29.4%)	170 (73.0%)	63 (27.0%)		3.36 ± 1.30	
Non	185 (23.3%)	147 (79.5%)	38 (20.5%)		3.34 ± 0.99	
Lymphovascular invasion						
Negative	705 (88.9%)	520 (73.8%)	185 (26.2%)	0.266*	3.41 ± 1.09	0.397†
Positive	88 (11.1%)	60 (68.2%)	28 (31.8%)		3.51 ± 1.33	
T stage						
T1	296 (37.3%)	247 (83.4%)	49 (16.6%)	0.000*	3.16 ± 0.97	<0.001†
T2	202 (25.5%)	146 (72.3%)	56 (27.7%)		3.38 ± 1.16	
T3	204 (25.7%)	125 (21.3%)	79 (38.7%)		3.69 ± 1.29	
T4a	91 (11.5%)	62 (68.1%)	29 (31.9%)		3.70 ± 1.18	
pN stage						
N0	569 (71.8%)	424 (74.5%)	145 (25.5%)	0.163*	3.39 ± 1.15	0.040†
N(1‐3a)	224 (28.2%)	156 (69.6%)	68 (30.4%)		3.50 ± 1.25	
AJCC stage						
IA	261 (32.9%)	219 (83.9%)	42 (16.1%)	<0.001*	3.17 ± 0.98	<0.001†
IB	135 (17.0%)	97 (71.9%)	38 (28.1%)		3.33 ± 1.19	
IIA	156 (19.7%)	105 (67.3%)	51 (32.7%)		3.58 ± 1.31	
IIB	241 (30.4%)	159 (66.0%)	82 (34.0%)		3.61 ± 1.18	
Pretreatment Anemia						
Anemia	175 (22.1%)	104 (59.4)	71 (40.6%)	<0.001*	3.75 ± 1.65	<0.001†
Normal	618 (77.9%)	476 (77.0%)	142 (23.0%)		3.37 ± 1.06	
Blood platelet						
<300/L	660 (83.2%)	514 (77.9%)	146 (22.1%)	<0.001*	3.34 ± 1.06	<0.001†
≧300 g/L	133 (16.8%)	66 (49.6%)	67 (50.4%)		4.00 ± 1.40	

IQR, interquartile range; PFC, plasma fibrinogen concentration; PFC0, plasma fibrinogen concentration <4 g/L; PFC1, plasma fibrinogen concentration ≧4 g/L.

* Chi‐squared test.

^†^ Kruskal‐Wallis or Mann‐Whitney *U* test.

### 3.2 PFC level as an independent prognostic indicator in patients with stage I‐II GC

The 5‐year OS and CSS rates of the 793 patients with stage I‐II GC were 79.4% and 81.5%, respectively. Multivariate Cox regression analysis revealed that PFC level was an independent risk factor for OS (HR = 1.7; 95% CI = 1.3‐2.3; *P* < 0.001), CSS (HR = 2.1; 95% CI = 1.5‐3.0; *P* < 0.001), and advanced AJCC‐TNM stage. The results are summarized in Table 2.

**Table 2:** Univariate and multivariate survival analysis of the prognostic factors for stage I‐II gastric cancer patients

Factors	Overall survival				Cancer specific survival			
	Univariate		Multivariate		Univariate		Multivariate	
	Survival (5 y), %	*P*	HR (95% CI)	*P*	Survival (5 y), %	*P*	HR (95% CI)	*P*
Age								
<60 y	83.5	<0.001	1	0.003	85.6	0.009	1	0.181
≧60 y	74.1		1.5 (1.2‐2.1)		79.8		1.3 (0.9‐1.7)	
Gender								
Male	79.8	0.612			83.9	0.439		
Female	78.5				80.9			
Tumor location								
Upper	72.3	0.075			74.5	0.129		
Middle‐Lower	80.2				83.7			
Tumor size								
<5 cm	83.5	<0.001	1	0.158	86.5	<0.001	1	0.086
≧5 cm	68.9		1.2 (0.9‐1.7)		74.0		1.3 (0.9‐1.9)	
AJCC stage								
IA	95.8	<0.001	1		97.7	<0.001	1	
IB	81.5		2.4 (1.4‐4.2)	0.002	83.5		4.7 (2.2‐10.2)	<0.001
IIA	73.7		3.4 (2.0‐5.8)		78.4		7.1 (3.3‐14.8)	<0.001
IIB	64.3		5.3 (3.2‐8.6)	<0.001	69.8		10.8 (5.3‐21.8)	<0.001
eLN								
≧16	80.4	0.036	1		84.2	0.083	1	
<16	73.5		1.8 (1.2‐2.6)	0.002	76.6		1.8 (1.2‐2.8)	0.004
LVI								
No	80.4	0.235			84.1	0.049		
Yes	71.6				74.8			
Blood transfusion								
No	80.6	0.070			83.3	0.132		
Yes	68.8				76.2			
Blood platelet								
<300	80.3	0.288			83.8	0.224		
≧300	75.2				79.3			
Anemia								
No	82.2	<0.001	1		84.6	0.015	1	
Yes	69.7		0.9 (0.7‐1.2)	0.544	77.6		1.2 (0.8‐1.8)	0.326
PFC stage								
PFC0	84.6	<0.001	1		88.3	<0.001	1	
PFC1	65.3		1.7 (1.3‐2.3)	<0.001	68.7		2.1 (1.5‐3.0)	<0.001

eLN, Number of examined lymph node; CI, confidence interval; HR, hazard ratio; LVI, Lymphovascular invasion; PFC0, plasma fibrinogen concentration <4 g/L; PFC1, plasma fibrinogen concentration ≧4g/L.

### 3.3 Analysis of prognosis of patients with stage I‐II GC after incorporation of the PFC level into the AJCC staging system

**Figure 1:**
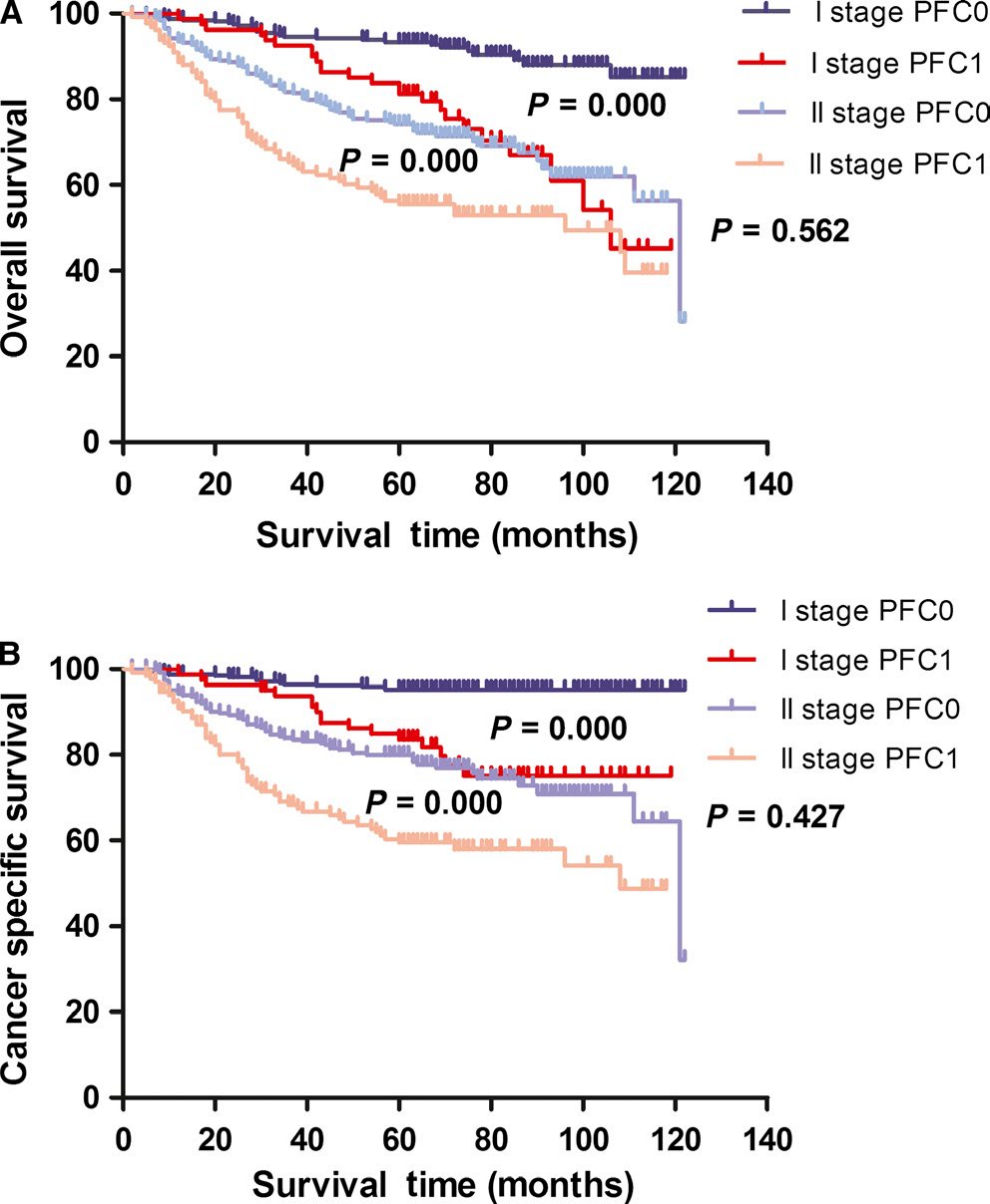
Kaplan‐Meier curves for OS and CSS according to PFC level in GC patients with TNM stage I and II. Patients with TNM stage I‐PFC1 and TNM stage II‐PFC0 had a similar OS (A: *P* = 0.562) and CSS (B: *P* = 0.427). OS, overall survival; CSS, cancer‐specific survival; PFC0, plasma fibrinogen concentration <4 g/L; PFC1, plasma fibrinogen concentration ≧4 g/L

**Figure 2:**
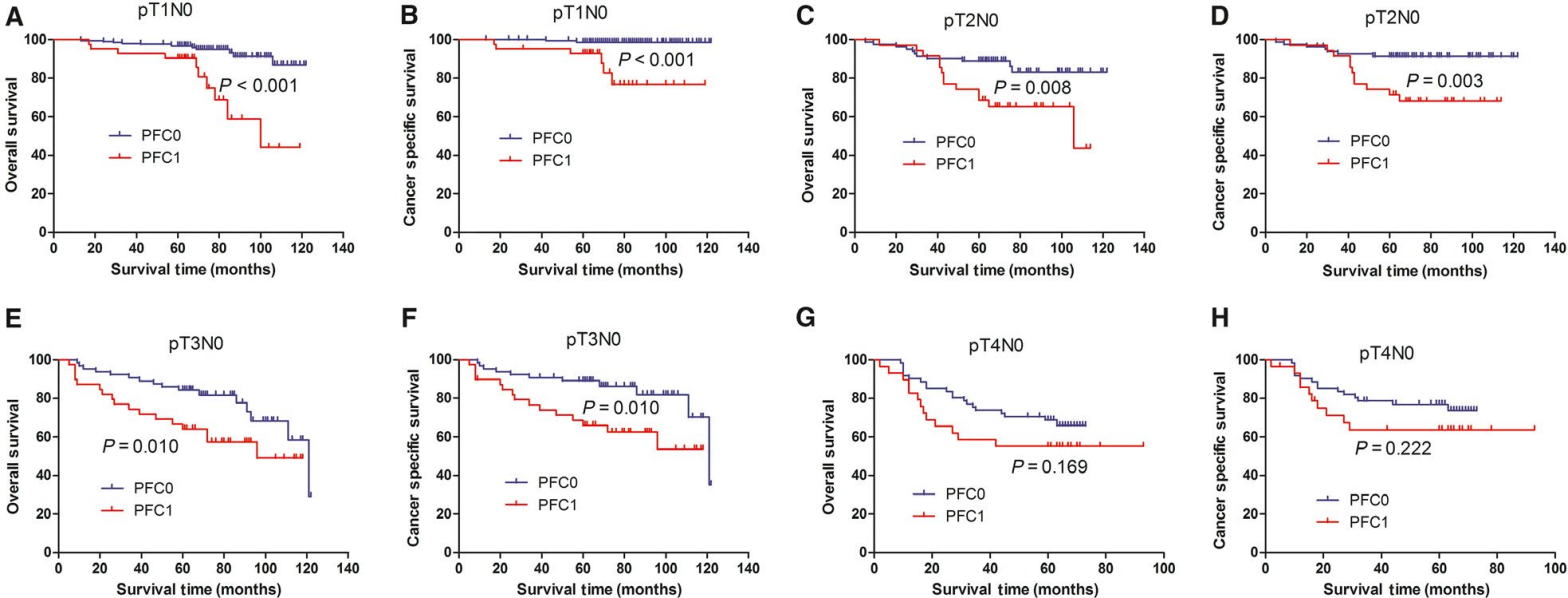
Kaplan‐Meier curves for OS and CSS according to PFC level in N0 patients. For patients with node‐negative GC, a significant survival difference was found in those with T1, T2 and T3 stage (A–F), but not in those with T4a (G, H). OS, overall survival; CSS, cancer specific survival; PFC0, plasma fibrinogen concentration <4 g/L; PFC1, plasma fibrinogen concentration ≧4 g/L

**Figure 3:**
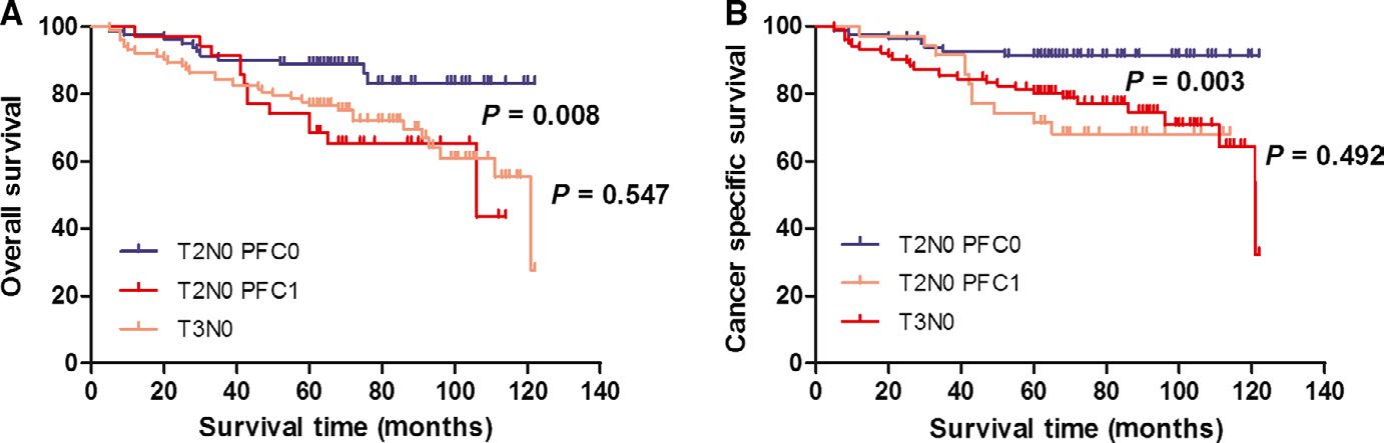
Kaplan‐Meier curves for OS and CSS according to PFC level in T2 and T3 patients. Patients with T2N0‐PFC1 and T3N0 had similar OS (A: *P* = 0.547) and CSS (B: *P* = 0.492). OS, overall survival; CSS, cancer‐specific survival; PFC0, plasma fibrinogen concentration, <4 g/L; PFC1, plasma fibrinogen concentration ≥4 g/L

We incorporated the PFC level into different AJCC stages to investigate its prognostic capacity in patients with stage I‐II GC. Consequently, new groups were formed based on the PFC level and AJCC stage, such as TNM stage I‐PFC0 and TNM stage I‐PFC1, TNM stage II‐PFC0 and TNM stage II‐PFC1, pN0‐PFC0 and pN0‐PFC1, and pT1N0‐PFC0 and pT1N0‐PFC1, among others, as shown in Table 3. The 5‐year OS and CSS of the various AJCC and PFC stage combinations were calculated to compare the survival curves of the corresponding groups via Kaplan‐Meier method. The results showed that the prognosis of patients in the TNM I and II stage and pN0 and pN (+) stage can be excellently stratified according to the PFC level. In addition, the significant survival difference of the same AJCC stage PFC1 was adequate to compensate with the late AJCC stage‐PFC0. For example, the survival of patients with TNM I stage‐PFC1 was similar with that of patients with TNM II stage‐PFC0 (Figure 1A,B), and similar findings were observed in those with pN0 and pN(+). For patients with node‐negative GC, a significant survival difference was found in those with T1, T2, and T3 stage, but not in those with T4a (Figure 2A‐H). The prognosis for patients with T2N0‐PFC1 GC was similar with those with T3N0 (Figure 3C,D).

**Table 3:** Overall and cancer‐specific survival of i‐ii stage gastric cancer of AJCC stage groups after incorporation of PFC stage

Stage group	N	Overall survival			Cancer specific survival		
		Survival (5 y), %	HR	*P*	Survival (5 y), %	HR	*P*
TNM I‐PFC0	316	93.3	1	<0.001	95.2	1	<0.001
TNM I‐PFC1	80	83.3	3.4		83.6	4.5	
TNM II‐PFC0	264	74.2	1	0.001	80.0	1	<0.001
TNM II‐PFC1	133	55.6	1.7		59.5	1.9	
N0‐PFC0	424	89.4	1	<0.001	92.9	1	<0.001
N0‐PFC1	145	71.0	2.9		74.6	3.8	
T1N0‐PFC0	218	95.1	1	<0.001	98.6	1	<0.001
T1N0‐PFC1	42	89.8	3.7		92.8	3.3	
T2N0‐PFC0	81	82.9	1	0.008	91.2	1	0.003
T2N0‐PFC1	35	64.3	2.0		71.4	3.7	
T3N0‐PFC0	64	73.6	1	0.010	89.0	1	0.010
T3N0‐PFC1	39	54.4	1.7		66.0	2.7	
T4aN0‐PFC0	61	69.3	1	0.169	76.9	1	0.222
T4aN0‐PFC1	29	55.2	1.6		59.9	1.6	

HR, hazard ratio; PFC0: plasma fibrinogen concentration <4 g/L; PFC1: plasma fibrinogen concentration ≧4 g/L,N(+):N stage 1‐3a.

### 3.4 Multivariate survival analysis to investigate the independent prognostic significance of PFC stage in patients with TNM stage I and T1‐4aN0 GC

Of the 396 patients with stage I GC and the 569 patients with stage T1‐4aN0 GC, 140 and 244 received postoperative oral or intravenous adjuvant chemotherapy, respectively. To determine the independent prognostic factors for stage I and T1‐4aN0 patients, we used the Cox regression model to analyze the prognostic significance of PFC level in all patients with stage I and T1‐4aN0 GC and in those who did not receive adjuvant chemotherapy.

Our results showed that PFC stage and AJCC‐TNM stage were independent prognostic factors for patients with stage I and T1‐4aN0 GC regardless of the presence or absence of adjuvant chemotherapy. The results are presented in Tables 4 and 5.

**Table 4:** Multivariate survival analysis of the prognostic factors for all stage I and N0 gastric cancer patients

Factors	Stage I (n = 396)				Stage T1‐4aN0 (n = 569)			
	Overall survival		Cancer specific survival		Overall survival		Cancer specific survival	
	HR (95% CI)	*P*	HR (95% CI)	*P*	HR (95% CI)	*P*	HR (95% CI)	*P*
Age								
≧60 y vs <60 y	1.7 (0.9‐3.1)	0.065	1.1 (0.5‐2.3)	0.791	1.4 (0.9‐2.1)	0.088	1.0 (0.6‐1.6)	0.969
Tumor size								
<5 cm vs ≧5 cm	1.2 (0.6‐2.5)	0.600	1.7 (0.7‐3.9)	0.199	1.4 (0.9‐2.1)	0.135	1.4 (0.9‐2.4)	0.124
AJCC stage								
IB vs IA	2.2 (1.2‐4.1)	0.008	4.5 (2.0‐9.9)	<0.001	2.2 (1.2‐4.0)	0.009	4.2 (1.8‐9.5)	<0.001
IIA vs IA					3.0 (1.7‐5.4)	<0.001	6.4 (2.9‐14.1)	<0.001
IIB vs IA					5.9 (3.3‐10.6)	<0.001	11.0 (5.0‐24.7)	<0.001
eLN								
<16 vs ≧16	2.2 (1.2‐4.1)	0.008	2.6 (1.2‐5.6)	0.012	1.6 (0.9‐2.4)	0.054	1.7 (0.9‐2.9)	0.063
Anemia								
Yes vs no	1.3 (0.6‐2.6)	0.309	1.3 (0.5 −3.0)	0.589	1.3 (0.8‐2.0)	0.273	1.9 (1.1 −3.3)	0.022
PFC stage								
PFC1 vs PFC0	2.5 (1.4‐4.3)	0.003	3.0 (1.4‐6.1)	0.004	2.2 (1.5‐3.2)	<0.001	3.0 (1.9‐4.8)	0.000

eLN, Number of examined lymph node; CI, confidence interval; HR, hazard ratio; PFC0, plasma fibrinogen concentration <4 g/L; PFC1, plasma fibrinogen concentration ≧4 g/L.

**Table 5:** Multivariate survival analysis of the prognostic factors for stage I and N0 gastric cancer patients without adjuvant chemotherapy

Factors	Stage I (n = 256)				Stage T1‐4aN0 (n = 325)			
	Overall survival		Cancer specific survival		Overall survival		Cancer specific survival	
	HR (95% CI)	*P*	HR (95% CI)	*P*	HR (95% CI)	*P*	HR (95% CI)	*P*
Age								
≧60 y vs <60 y	2.5 (1.1‐5.6)	0.032	1.6 (0.5‐5.4)	0.441	1.4 (0.8‐2.5)	0.191	1.0 (0.5‐1.9)	0.929
Tumor size								
<5 cm vs ≧5 cm	0.7 (0.2‐2.2)	0.503	1.2 (0.3‐4.4)	0.816	1.0 (0.6‐1.8)	0.956	1.1 (0.5‐2.4)	0.741
AJCC stage								
IB vs IA	2.5 (1.1‐5.5)	0.023	4.5 (2.0‐9.9)	<0.001	2.8 (1.3‐5.9)	0.008	4.2 (1.5‐12.3)	0.008
IIA vs IA					5.0 (2.4‐10.2)	<0.001	10.1 (3.8‐27.2)	<0.001
IIB vs IA					15.8 (7.6‐33)	<0.001	27.6 (9.9‐75.6)	<0.001
eLN								
<16 vs ≧16	2.0 (0.9‐4.3)	0.094	3.2 (1.1‐9.7)	0.039	1.6 (0.9‐2.9)	0.083	1.7 (0.8‐3.6)	0.186
Anemia								
Yes vs no	1.3 (0.5‐3.9)	0.591	0.9 (0.2 −3.5)	0.840	2.1 (1.1‐4.0)	0.025	3.3 (1.4 −8.0)	0.008
PFC stage								
PFC1 vs PFC0	3.2 (1.5‐6.9)	0.003	4.6 (1.5‐14)	0.004	2.4 (1.4‐4.1)	0.001	3.2 (1.6‐6.4)	0.001

eLN, Number of examined lymph node; CI, confidence interval; HR, hazard ratio; PFC0, plasma fibrinogen concentration <4g/L; PFC1, plasma fibrinogen concentration ≧4g/L.

For the 140 stage I patients administered adjuvant chemotherapy, PFC stage was not an independent prognostic factor for OS (*P* = 0.640, HR = 1.256) and CSS (*P* = 0.263, HR = 1.804). By contrast, PFC stage was an independent prognostic factor for OS (*P* = 0.049, HR = 1.904) and CSS (*P* = 0.004, HR = 2.756) among the 244 N0 patients administered adjuvant chemotherapy.

### 3.5 Difference in types of recurrence between PFC0 patients and PFC1 patients

Of the 396 patients with stage I GC and the 397 patients with stage II GC, 32 and 118 developed recurrence or metastasis after curative resection, respectively. Among the stage I GC patients with recurrence or metastasis, the occurrence rates of hematogenous metastasis, lymph node metastasis, and peritoneal implantation metastasis were 33.3% (5/15), 66.7% (10/15), and 33.3% (5/15), respectively, for the PFC0 patients and 64.7% (11/17), 35.3% (6/17), and 41.2% (7/17), respectively, for the PFC1 patients. In the stage II GC patients with recurrence or metastasis, the occurrence rates of hematogenous metastasis, lymph node metastasis, and peritoneal implantation metastasis were 55.6% (35/63), 34.5% (22/63), and 49.2% (31/63), respectively, for PFC0 and 61.8% (34/55), 27.3% (15/55), and 45.6% (25/55), respectively, for PFC1.

### 3.6 Calculation of the C‐index to evaluate the capability of PFC in improving the predictive accuracy of the AJCC stage for patients with stage I and T1‐4aN0 GC

Finally, we studied whether the combination of PFC level with the present AJCC‐TNM stage would improve the predictive accuracy for patients with stage I and T1‐4aN0 GC. We compared the accuracy of different models with and without the combination of the PFC level to the AJCC‐TNM stage to calculate Harrell's C‐index. In terms of OS of patients with stage I GC, the C‐index of the AJCC‐TNM staging model (IA and IB) was 0.659, but it improved to 0.702 when the PFC level was combined with the AJCC‐TNM staging. Similarly, in patients with T1‐4aN0 GC, the C‐index of the model including the TNM stage (IA, IB, IIA, and IIB) and PFC stage was 0.748, but it decreased to 0.714 when the PFC level was excluded in the model, and the analysis was based on T stage alone. A similar result was achieved for CSS. The C‐index increased from 0.688 to 0.735 in patients with stage I GC and from 0.730 to 0.769 in patients with T1‐4aN0 when the PFC stage was added to the AJCC staging system. The results are presented in Table 6.

**Table 6:** Comparison of the prognostic accuracy of AJCC‐TNM staging system and AJCC‐TNM + PFC staging system in GC with stage I and N0

		Stage I		pT1‐4aN0	
		TNM	TNM + PFC	TNM	TNM + PFC
OS analysis	c‐ index	0.659	0.702	0.714	0.748
CSS analysis	c‐ index	0.688	0.735	0.730	0.769

c‐ index, concordance index; OS, overall survival; CSS, cancer‐specific survival; PFC1, plasma fibrinogen concentration.

## 4 DISCUSSION

Abnormal coagulation and fibrinolysis frequently occur in patients with malignant tumors. As a kind of glycoprotein synthesized in the hepatocyte, fibrinogen (also known as clotting factor I) plays an important role in blood coagulation and the platelet accumulation. During the end of the clotting process, fibrinogen is converted enzymatically by thrombin to fibrin and subsequently to a fibrin‐based blood clot. Recently, several reports suggested that elevated preoperative fibrinogen was associated with the progression and prognosis of malignancies, including lung,^14^ cervical,^15^ breast,^16^ pancreatic,^17^ esophageal,^18^ prostate,^19^ renal cell,^20^ and colon^21^ cancers. Previous studies have shown that high preoperative fibrinogen is associated with poor survival in GC patients who underwent curative gastrectomy, but these studies enrolled all stage I‐III or I‐IV patients, and no study had focused on a subgroup analysis in stage I and N0 patients.

Our study analyzed the OS and CSS of patients with stage I‐II GC after the preoperative PFC level was incorporated into the AJCC staging system. The results indicated that the PFC level is a robust prognostic indicator and showed excellent prognostic discriminatory capability for each AJCC stage expect for T4aN0. Specifically, we noticed a substantial survival difference in TNM I stage‐PFC0 and TNM I stage‐PFC1, T2N0‐PFC0, and T2N0‐PFC1. The result is helpful in developing postoperative treatment plans for patients with stage I GC. The therapeutic benefit of postoperative adjuvant therapy in patients with stage I GC is controversial due to the lack of RTC research on patients with stage I GC. Moreover, the National Comprehensive Cancer Network guideline only recommends postoperative adjuvant therapy for patients with T1N1 and high‐risk T2N0. The high‐risk factors include poorly differentiated or high‐grade cancer, lymphovascular invasion, neural invasion, age <50 years, and partial resection of the D2 lymph node. We observed that patients with pT2N0‐PFC1 and T3N0, TNM stage I‐PFC1, and TNM stage II‐PFC0 had similar prognosis. These results suggested that PFC ≧4.0 g/L may be a high‐risk factor for poor prognosis in patients with stage I GC after radical gastrectomy.

To support the above conclusion, we investigated the prognostic value of PFC with a focus on patients with stage I and T1‐4aN0. To exclude the effect of postoperative chemotherapy on the survival of patients with stage I and N0, we constructed multiple Cox regression models for all patients with stage I and N0 or the patients with stage I and N0 who underwent postoperative chemotherapy. Cox models revealed that PFC was an independent risk factor for poor prognosis in patients with stage I and T1‐4aN0 regardless of whether or not adjuvant chemotherapy was administered. In terms of the capability of PFC level to improve the predictive accuracy of AJCC‐TNM staging system, the C‐index indicated that the combination of PFC level with the AJCC‐TNM staging can improve the latter's accuracy for predicting survival in patients with stage I and T1‐4aN0 GC. Several studies showed that the T stage alone is inadequate for predicting the survival of node‐negative patients.^22^, ^23^, ^24^ In the current study, we proved that PFC level was an independent indicator for survival and can improve the accuracy of predicting prognosis when combined with the T stage.

Currently, the mechanisms for the negative effect of elevated PFC on the prognosis of GC remain unknown. Several potential mechanisms were demonstrated in other malignant tumors. Palumbo et al^25^ reported that fibrinogen is favorable for the formation of tumor stroma and adhesion of circulating tumor cells in the vasculature, thus increasing the probability of embolic tumor metastasis. In addition, fibrinogen can bind some growth factors, such as vascular endothelial growth factor and contribute to the angiogenesis of tumor tissues that leads to tumor growth and metastasis.^26^, ^27^ Third, as a multiple integrin and nonintegrin receptors in tumor cells, fibrinogen can mediate cellular interactions and command tumor cell activities, including proliferation, migration, and apoptosis.^28^, ^29^, ^30^ Based on these mechanisms, we speculated that elevated PFC might contribute to hematogenous metastasis of tumor cell. Interestingly, the differences in types of recurrence between PFC0 patients and PFC1 patients in the current study support this hypothesis. The results indicated that the incidence of hematogenous metastasis after curative surgery was higher in patients with PFC1 than that in PFC0.

Although we performed a subgroup survival analysis of PFC level in stage I‐II CG, there are still several limitations in the study. First, we did not acquire information on inflammatory factors, such as leukocyte and C‐reactive protein, which may influence PFC and the prognosis of GC patients and we could not eliminate the patients with potentially inflammatory disease. Moreover, we did not use X‐tile program to determine the optimal cut‐off values for fibrinogen and used the reference standard recommended by the manufacturer. By “reference standard,” PFC of 2‐4.0 g/L was considered as “normal” in the study, which is also the established standard, making it convenient for clinicians to estimate fibrinogen level. In addition, although a large number of patients with stage I‐II were included in the study, because we focused on some subgroup analysis (such as “TNM stage I”“TNM stage T2N0”), as a result, the sample size is insufficient for validation cohort analysis. Finally, some potential biases due to the study being conducted in single institution cannot be excluded. Thus, a multicenter and controlled study is needed to validate our results.

## 5 CONCLUSIONS

Our results indicated that preoperative PFC is a prognostic factor that can be used to supplement the accuracy of the current TNM staging for predicting the prognosis of patients with stage I‐II GC and to help making postoperative treatment plans (chemotherapy or follow‐up observation). Moreover, fibrinogen‐related therapies may be a novel approach to improve survival rate of GC.^31^ The good news is studies have shown that anticoagulant treatment has antitumor effects in vivo and in vitro.^32^

## CONFLICT OF INTERESTS

All authors have no conflict of interest to declare.

## ETHICS STATEMENT

The study protocol was approved by the Research Ethics Committee of China Medical University (Shenyang, China), and all study procedures were in accordance with the Declaration of Helsinki and its later amendments.
